# Unmasking Photophobia and Blepharospasm Mechanisms: The Role of Optic Chiasm and Trigeminal Nerve Compression in a Giant Pituitary Adenoma Case

**DOI:** 10.7759/cureus.91148

**Published:** 2025-08-27

**Authors:** Ayaka Hasegawa, Yoshiaki Tagawa, Yasuhiro Shinmei, Akihiro Shinkai, Susumu Ishida

**Affiliations:** 1 Department of Ophthalmology, Tokeidai Memorial Hospital, Sapporo, JPN; 2 Department of Ophthalmology, Hokkaido University, Sapporo, JPN

**Keywords:** blepharospasm, giant pituitary adenoma, optic chiasm compression, photophobia, trigeminal nerve compression

## Abstract

Blepharospasm, characterized by abnormal blinking and sensory hypersensitivity such as photophobia and ocular pain, is thought to arise from pathological sensorimotor integration. We report a rare case of a male teenager with a growth hormone-secreting giant pituitary adenoma compressing both the optic chiasm and bilateral trigeminal nerves. Initially, the patient presented with visual disturbances and bitemporal hemianopia, without photophobia or ocular pain. Following partial tumor resection, which relieved compression of the optic chiasm but not the trigeminal nerves, he developed severe photophobia, deep ocular pain, and bilateral secondary blepharospasm. These symptoms persisted despite pharmacologic interventions, including pregabalin and topical rebamipide. A second surgery, which decompressed the trigeminal nerves, led to the complete resolution of all symptoms. The clinical course suggests that trigeminal nerve compression, particularly of the ophthalmic branch (V1), may induce hypersensitivity states resulting in neuropathic pain, photophobia, and secondary blepharospasm. We hypothesize that initial optic chiasm compression suppressed photophobia by disrupting non-visual photophobia circuits through the suprachiasmatic nuclei to pulvinar nuclei. After decompression, restoration of this pathway unmasked the hypersensitivity induced by ongoing trigeminal compression. The complete resolution of symptoms following trigeminal decompression supports the role of peripheral sensory nerve compression in the pathogenesis of photophobia and secondary blepharospasm. This case provides novel clinical evidence for the interrelationship between the trigeminal and visual pathways in sensorimotor disorders.

## Introduction

Blepharospasm is characterized by abnormal blinking control, often accompanied by sensory hypersensitivity such as photophobia and ocular pain. Sensory and motor functions are interrelated, forming a sequence of movements called sensorimotor integration. Due to this sensorimotor integration, sensory and motor abnormalities are also interrelated [1]. In blepharospasm, photophobia and difficulty in opening eyelids form a vicious cycle that causes the symptoms.

Photophobia involves abnormalities in visual and nociceptive pathways via the optic and trigeminal nerves. The pathway via the optic nerve can be further divided into visual and non-visual information. Unlike the visual information pathway, which processes light stimuli to recognize objects, colors, and brightness, the non-visual information pathway is a circuit that regulates circadian rhythms, arousal levels, and mood in response to light [2]. Recent studies on migraine have identified a pathway involving the optic nerve, optic chiasm, suprachiasmatic nucleus, and pulvinar nucleus as one of the circuits responsible for photophobia [3,4]. Notably, the pulvinar nucleus of the thalamus has been reported to contain cells that respond to both nociceptive and photic stimuli [5].

It has been known that cases of optic chiasm compression caused by pituitary tumors can result in photophobia [6]. However, the mechanisms underlying photophobia due to optic chiasm compression remain unclear.

We encountered a case of a giant pituitary adenoma compressing the optic chiasm and bilateral trigeminal nerves. Following partial tumor resection, which decompressed the optic chiasm, the patient developed ocular pain, photophobia, and secondary blepharospasm. These symptoms resolved after an additional resection of the residual tumor, compressing the trigeminal nerves. We discuss this clinical course, incorporating insights from previous reports. This case provides novel insights into the mechanism by which a tumor near the optic chiasm induces photophobia and serves as a valuable clinical example demonstrating the involvement of the trigeminal and optic nerves-particularly the non-visual photic pathway-in the pathogenesis of photophobia.

## Case presentation

The patient was a male teenager who presented with a three-year history of headaches. He was referred to our department for preoperative evaluation after being diagnosed with a pituitary tumor on magnetic resonance imaging (MRI) and acromegaly based on facial features by a neurosurgical department. Comprehensive evaluations confirmed the diagnosis of a growth hormone-secreting pituitary adenoma. The patient had no significant medical or family history. At least, there was no family history or previous history of migraine.

**Figure 1:**
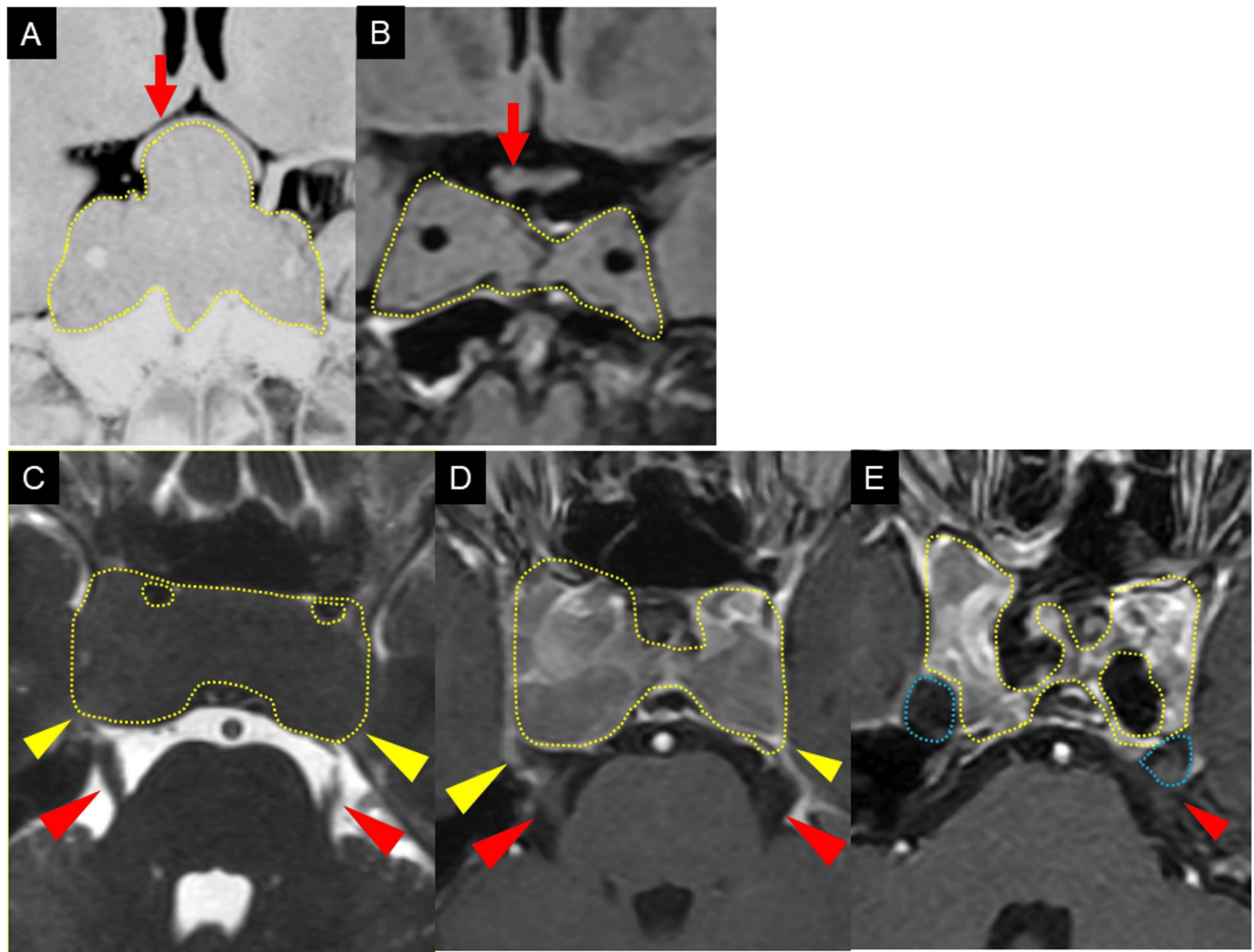
Magnetic resonance imaging (MRI) before surgery, after the first surgery, and after the second surgery Yellow dashed line: pituitary tumor; blue dashed line: Meckel's cave; red arrow: optic chiasm; red arrowhead: trigeminal nerve; yellow arrowhead: bone/dura (A) Preoperative coronal view showing strong optic chiasm compression and deformation by the pituitary tumor. At that time, no symptoms were present. (B) Postoperative coronal view (first surgery) showing resolved optic chiasm compression. Postoperatively, the patient developed photophobia, ocular pain, and difficulty opening the eyelids. (C) Preoperative axial view showing Meckel's cave obliteration by the pituitary tumor and presumed trigeminal nerve compression. At that time, no symptoms were present. (D) Postoperative axial view (first surgery) showing Meckel's cave obliteration and still presumed trigeminal nerve compression. Postoperatively, the patient developed photophobia, ocular pain, and difficulty opening the eyelids. (E) Postoperative axial view (second surgery) showing restored Meckel's cave and relieved trigeminal nerve compression. Following the second surgery, photophobia, ocular pain, and difficulty opening the eyelids resolved completely.

**Figure 2:**
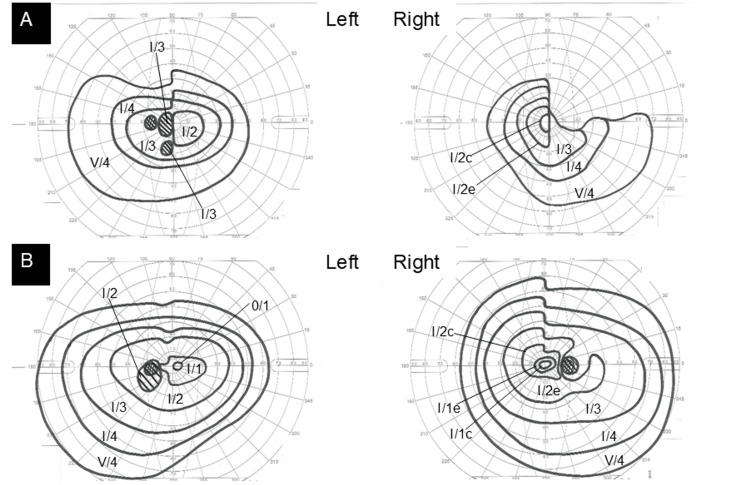
Visual field tests before and after the first surgery (A) Preoperative visual field tests for the right and left eyes, respectively, showing bitemporal hemianopia. (B) Postoperative visual field tests for the right and left eyes, respectively, showing significant improvement in bitemporal hemianopia.

At the initial visit, his best-corrected visual acuity was 0.4 (right) and 0.7 (left), with normal intraocular pressures. Both eyes had normal anterior and posterior segments. The optic nerves in both eyes were well-defined at the margins, with no swelling or redness, and were normal. Preoperative MRI revealed a giant pituitary tumor compressing and significantly deforming the optic chiasm (Figure 1A). Because the pituitary tumor was large, the surrounding dura mater and bone were compressed from the inside. The tumor occupied Meckel's cave, which was poorly depicted; the trigeminal nerve entering this region seemed to be in contact with the tumor (Figure 1C). The eye position was normal in both eyes, there were no restrictions in eye movement, and no abnormalities in the pupils or ptosis were observed, so we recognized that the cranial nerves controlling the eye muscles were not problematic. Goldman perimetry revealed bitemporal hemianopia (Figure 2A).

Partial tumor resection was performed three months after the initial visit to relieve optic chiasm compression and normalize insulin-like growth factor 1 (IGF-1) levels, as whole tumor resection extending into the cavernous sinus was deemed high-risk. Postoperatively, optic chiasm compression was resolved (Figure 1B); however, the tumor was still thought to compress the trigeminal nerve (Figure 1D). The best-corrected visual acuity of both eyes improved to 1.0. Bilateral temporal hemianopia improved almost completely one month after the initial surgery (Figure 2B). The eyes still showed slight hemianopia, but this resolved on visual field testing one year later. However, photophobia, deep ocular pain, and bilateral abnormal blinking developed shortly after surgery. These postoperative symptoms persisted beyond the perioperative period and gradually worsened for eleven months. The severity of blepharospasm was classified as Grade 2 on a 5-point scale based on the voluntary blinking test [7]. According to the Jankovic Rating Scale, the severity score was 1 (range: 0-4), and the frequency score was also 1 (range: 0-4) [8]. Given the positive voluntary blinking tests, strong photophobia, and the progressive worsening of symptoms, the condition was diagnosed as coexisting secondary bilateral blepharospasm rather than transient postoperative symptoms. Differential diagnoses included essential blepharospasm, trigeminal neuralgia, and migraine-associated photophobia due to the similarity of symptoms. However, considering the onset in relation to pituitary tumor surgery, the chronic persistence of symptoms without identifiable exacerbating factors, and the clinical course and family history, these conditions were deemed unlikely.

To suppress growth hormone and IGF-1 levels, intramuscular injections of the somatostatin analogue octreotide (30 mg) were administered every four weeks [9]. A modest reduction in ocular pain and photophobia was observed for approximately two weeks following each injection; however, the therapeutic effect was insufficient. Corneal sensitivity was assessed using the Cochet-Bonnet esthesiometer [10]. After determining the threshold for tactile sensation, the filament length was gradually shortened in 5 mm increments until the patient reported pain; this point was defined as the pain threshold [11]. In both eyes, corneal tactile sensitivity was measured at 60 mm, and the pain threshold was 50 mm, indicating ocular hyperalgesia. Topical anesthesia was administered to the patient for evaluative purposes, resulting in relief of deep ocular pain, while photophobia persisted [12]. These findings suggested a central origin of the ocular pain; however, peripheral stimuli might also contribute to symptom exacerbation. Two percent rebamipide eye drops, an approved therapeutic agent for dry eye disease in Japan [13], were employed as a non-invasive and easily initiated treatment to mitigate nociceptive stimuli from the ocular surface. Topical administration of rebamipide four times daily resulted in a dramatic, albeit temporary, improvement in ocular pain, with the visual analog scale (VAS) score decreasing from 100 to 10. Corneal tactile sensitivity remained at 60 mm, while the corneal pain threshold decreased to 0 mm (5 mm stimulation did not cause pain). Although transient relief was achieved, the overall therapeutic effect was insufficient. Several months later, a gradual exacerbation of ocular pain was observed, with the VAS score reaching 60 mm. Suspecting that compression of sensory nerves might be contributing to the symptoms, treatment with pregabalin-a pharmacologic agent for neuropathic pain-was initiated [14]. Following oral pregabalin 75 mg twice daily administration, the VAS score improved markedly to 10 mm. Pregabalin was the most effective non-surgical treatment. However, the therapeutic effect of pregabalin diminished over time, and the VAS score eventually worsened again to 60 mm. Eleven months after the initial surgery, persistently elevated IGF-1 levels necessitated a second resection of the residual tumor.

**Figure 3:**
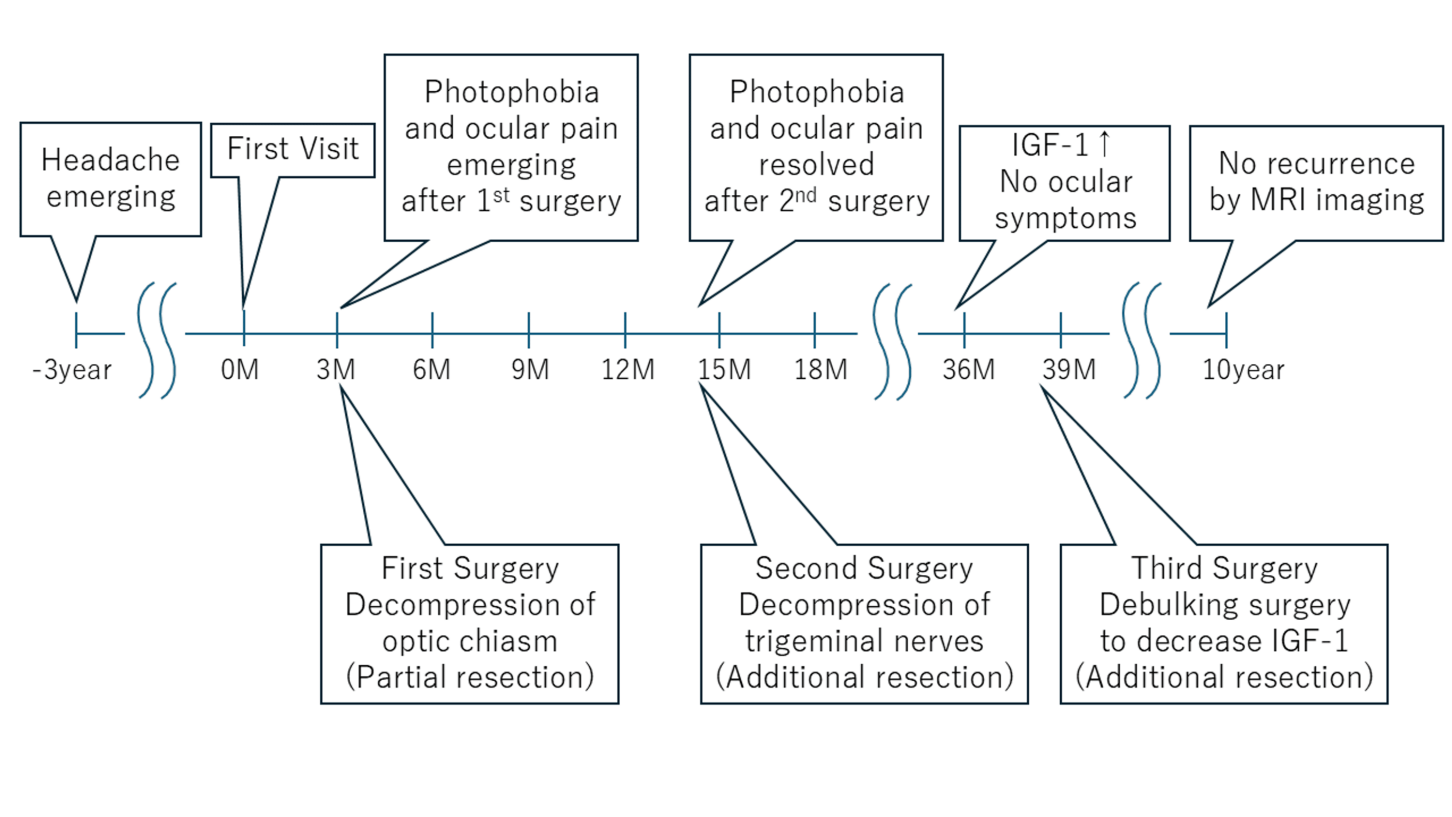
Timeline summarizing clinical events and interventions This figure illustrates the chronological sequence of clinical events and associated symptom changes in the present case. M: months since initial presentation; MRI: magnetic resonance imaging; IGF-1: insulin-like growth factor 1

MRI after the second surgery showed the partial residual tumor in the cavernous sinus; however, the trigeminal nerve was now visualized, indicating that most of the tumor in this region had been resected (Figure 1E). Following the second surgery, photophobia, ocular pain, and blinking abnormalities disappeared. No additional radiation therapy was performed, but secondary blepharospasm resolved completely, with no symptom recurrence. After that, although IGF-1 levels decreased, they did not return to normal, so cabergoline (a dopamine D2 receptor agonist) was added to the treatment regimen [15]. Cabergoline dosage was increased to address elevated IGF-1 levels, but auditory hallucinations emerged as a side effect, leading to additional tumor resection under MRI guidance two years later. During this period, there were no symptoms of photophobia, ocular pain, or blepharospasm. Subsequently, the patient has been followed up with an MRI for eight years, with no signs of recurrence, and IGF-1 levels are currently maintained within the normal range (Figure 3).

## Discussion

In this case, a pituitary adenoma seemed to stimulate the bilateral trigeminal nerve, causing photophobia and secondary blepharospasm. Strong compression on the optic chiasm appears to have masked these symptoms preoperatively.

Compression of the trigeminal nerve's ophthalmic branch (V1) can stimulate nociceptive pathways, leading to symptoms such as headache, deep ocular pain, photophobia, and blepharospasm-like manifestations. There is a previous case report of a trigeminal ganglioneuroma compressing the trigeminal nerve entering Meckel's cave, in which ocular pain and photophobia, like our case, appeared [16]. Furthermore, intense photophobia and ocular pain can induce reflexive blinking, which can cause abnormal blinking similar to blepharospasm. Symptom resolution after decompressing the trigeminal nerve during the second surgery supports the hypothesis that compression of the trigeminal V1 area induces hypersensitivity (neuropathic pain), leading to photophobia and secondary blepharospasm. The efficacy of pregabalin, a neuropathic pain agent, further supports this hypothesis. For instance, in the early stages of the disease, auditory schwannomas often cause trigeminal neuralgia by lightly contacting the trigeminal nerve. In contrast, they can strongly compress the trigeminal nerve in advanced stages, resulting in neuroparalytic keratopathy. Therefore, the nerve is known to exhibit hypersensitivity or insensitivity depending on the degree and duration of compression [17]. In this case, the tumor's contact with the trigeminal nerve on both sides likely caused hypersensitivity, resulting in photophobia, ocular pain, and eventually secondary bilateral blepharospasm due to pathological sensory-motor disintegration [1]. In this case, the patient's neuropathic ocular pain was presumed to be caused by compression of the trigeminal nerve. Notably, topical administration of rebamipide appeared to exert a partial therapeutic effect on the neuropathic pain. We have previously encountered several cases in which rebamipide eye drops seemed to alleviate neuropathic ocular pain [18,19], suggesting a potential role for this agent in such conditions. Further investigation is warranted to elucidate its efficacy and underlying mechanisms.

Photophobia is strongly linked to non-visual pathways via the suprachiasmatic nucleus. Tumors compressing the optic chiasm may alter photophobia thresholds. Migraine studies have shown that the pathway from the optic chiasm through the suprachiasmatic nuclei to the pulvinar nuclei is a key circuit for photophobia [3]. In our case, severe optic chiasm compression likely blocked this pathway, masking photophobia preoperatively. After initial decompression, the photophobia threshold normalized, unmasking symptoms. Conversely, Kawasaki and Purvin reported that a tumor lightly touching the optic chiasm caused severe photophobia [6]. Although these findings may appear contradictory, they can be interpreted as a difference in disease stage. In the photophobia circuit, as with other nerves, varying degrees and durations of compression can result in either hypersensitivity or hyposensitivity. Therefore, Kawasaki and Purvin's report can reflect the development of photosensitivity, while the present case can demonstrate suppression of photophobia symptoms due to the different ways of compression.

## Conclusions

This case highlights the role of trigeminal nerve compression in the development of photophobia and secondary blepharospasm. The temporal relationship between symptom onset and tumor decompression supports a mechanism of neuropathic hypersensitivity. Furthermore, severe optic chiasm compression may have suppressed photophobia via disruption of non-visual pathways, with the symptoms becoming apparent after decompression. These findings suggest a dual contribution of both trigeminal and optic pathways in symptom modulation, although further case series are needed to generalize the proposed mechanism. This case highlights the importance of re-evaluating with MRI in patients presenting with photophobia or blepharospasm-like symptoms, or in those with pituitary tumors who develop photophobia and ocular pain, to assess for compression of the optic chiasm or trigeminal nerve and consider surgical decompression if necessary.
